# The peroxisome proliferator-activated receptor delta +294T > C polymorphism and alcohol consumption on serum lipid levels

**DOI:** 10.1186/1476-511X-10-242

**Published:** 2011-12-23

**Authors:** Xian-Liang Wei, Rui-Xing Yin, Lin Miao, Dong-Feng Wu

**Affiliations:** 1 Department of Cardiology, Institute of Cardiovascular Diseases, the First Affiliated Hospital, Guangxi Medical University, 22 Shuangyong Road, Nanning 530021, Guangxi, People's Republic of China; 2 Department of Anatomy, School of Premedical Sciences, Guangxi Medical University, Nanning 530021, Guangxi, People's Republic of China

## Abstract

**Background:**

The single nucleotide polymorphism (SNP) of peroxisome proliferator-activated receptor delta (*PPARD*) gene affects serum lipid profiles, but to what extent alcohol consumption interferes with this association remains unknown. The present study was undertaken to compare the association of *PPARD *+294T > C (rs2016520) polymorphism and serum lipid levels in the nondrinkers and drinkers.

**Methods:**

A total of 685 unrelated nondrinkers and 497 drinkers aged 15-82 were randomly selected from our previous stratified randomized cluster samples. Genotyping of the *PPARD *+294T > C was performed by polymerase chain reaction and restriction fragment length polymorphism. Interactions of the *PPARD *+294T > C genotypes and alcohol consumption on serum lipid levels were detected by using a factorial regression analysis after controlling for potential confounders.

**Results:**

The levels of triglyceride (TG), high-density lipoprotein cholesterol (HDL-C), apolipoprotein (Apo) A1, and the ratio of ApoA1 to ApoB were higher in drinkers than in nondrinkers (*P *< 0.05-0.001). There were no significant differences in the levels of total cholesterol (TC), low-density lipoprotein cholesterol (LDL-C) and ApoB between the two groups (*P *> 0.05 for all). The frequencies of TT, TC and CC genotypes were 56.0%, 36.4% and 7.6% in nondrinkers, and 57.2%, 38.0% and 4.8% in drinkers (*P *> 0.05); respectively. The frequencies of T and C alleles were 74.2% and 25.8% in nondrinkers, and 76.2% and 23.8% in drinkers (*P *> 0.05); respectively. There was also no significant difference in the genotypic and allelic frequencies between males and females in both groups (*P *> 0.05 for all). The levels of TC in nondrinkers were different among the three genotypes (*P *= 0.01), the C allele carriers had higher serum TC levels than the C allele noncarriers. The levels of all seven lipid traits in drinkers were not different among the three genotypes (P > 0.05 for all). The interactions of *PPARD *+294T > C genotypes and alcohol consumption on serum lipid levels were not detected in the drinkers (*P >*0.05 for all). Multiple linear regression analysis showed that serum TC, HDL-C, LDL-C, ApoA1, and ApoB levels were correlated with genotypes in drinkers but not in nondrinkers (*P *< 0.05-0.01).

**Conclusions:**

These results suggest that the great majority of our study populations are beneficial from alcohol consumption. But there is no interaction between the *PPARD *+294T > C genotypes and alcohol consumption on serum lipid levels in the drinkers.

## Introduction

Abnormalities in lipid metabolism such as elevated total cholesterol (TC), triglyceride (TG), low-density lipoprotein cholesterol (LDL-C) and apolipoprotein (Apo) B levels, together with decreased high-density lipoprotein cholesterol (HDL-C), and ApoA1 levels, are considered as major risk factors for coronary artery disease (CAD) [1-4]. It is well known that serum lipid levels are modulated by multiple environmental and genetic factors and their interactions [5-11]. Numerous studies have evaluated the influence of alcohol consumption on CAD and serum lipid concentrations. Low to middle amounts of alcohol when taken on a regular basis have been shown to protect against CAD and death [12,13], whereas heavy drinking constitutes a severe risk condition. These results probably due in part to a dose-dependent increase in HDL-C and ApoA1 [14-17]. A decrease in LDL-C with increased alcohol intake has also been reported in some studies [18]. However, alcohol in doses > 30 g/day in both sexes can augment serum TG levels [15,19].

Peroxisome proliferator-activated receptors (PPARs) are ligand-activated nuclear hormone receptors that act as transcriptional regulators and are involved in glucose and lipid metabolism. Three different PPARs, PPAR-alpha (PPARA), PPAR-gamma (PPARG), and PPAR-delta (PPARD) have been characterized [20], and they are distinguished from each other by tissue distribution and cell activation. PPARA is mainly expressed in liver, muscle, kidney and heart, PPARG is most abundant in adipocytes, intestinal cells and macrophages and PPARD is expressed in many tissues [21-23]. PPARs are encoded by separate genes and characterized by distinct tissue and developmental distribution patterns. The *PPARD *gene was mapped to 6p21.2-p21.1 with 11 exons spanning 35 Kbp and is expressed ubiquitously [24]. The *PPARD *+294T > C (rs2016520) polymorphism in the 5'-untranslated region in exon 4 of the PPARD gene is located 87 nucleotides upstream of the start codon. It was shown that the single nucleotide polymorphism (SNP) influenced binding of Sp-1 resulting in higher transcriptional activity for the rare C allele than the common T allele [25]. Several previous studies have showed that the *PPARD *+294T > C polymorphism was associated with modifications of serum lipid concentrations in the general population and the risk of CAD [25-34] in dyslipidemic women and hypercholesterolemic men and cholesterol metabolites in Alzheimer's disease patients [35]. But the results are inconsistent in diverse populations [33,36]. Furthermore, little is known about the interactions of *PPARD *+294T > C polymorphism and alcohol consumption on serum lipid concentrations. Therefore, the aim of the present study was to compare the association of *PPARD *+294T > C (rs2016520) polymorphism and serum lipid levels in the nondrinkers and drinkers.

## Materials and methods

### Study population

A total of 685 unrelated nondrinkers and 497 drinkers were randomly selected from our previous stratified randomized cluster samples [5,6]. The age of the subjects ranged from 15 to 82 years, with an average age of 43.46 ± 16.50 years. All of the subjects were rural agricultural workers. The subjects with evidence of diseases related to atherosclerosis, CAD and diabetes have been excluded. None of them had been treated with β-adrenergic blocking agents and lipid-lowering drugs such as statins or fibrates. The present study was approved by the Ethics Committee of the First Affiliated Hospital, Guangxi Medical University. Informed consent was obtained from all subjects.

### Epidemiological survey

The survey was carried out using internationally standardized methods [37]. Information on demographics, socioeconomic status, and lifestyle factors was collected with standardized questionnaires. The alcohol information included questions about the number of liangs (about 50 g) of rice wine, corn wine, rum, beer, or liquor consumed during the preceding 12 months. Alcohol consumption was categorized into groups of grams of alcohol per day: < 25 and ≥ 25. Smoking status was categorized into groups of cigarettes per day: < 20 and ≥ 20. At the physical examination, several parameters including height, weight, and waist circumference were measured. Sitting blood pressure was measured three times with the use of a mercury sphygmomanometer after the subjects had a 5-minute rest, and the average of the three measurements was used for the level of blood pressure. Systolic blood pressure was determined by the first Korotkoff sound, and diastolic blood pressure by the fifth Korotkoff sound. Body weight, to the nearest 50 grams, was measured using a portable balance scale. Subjects were weighed without shoes and in a minimum of clothing. Height was measured, to the nearest 0.5 cm, using a portable steel measuring device. From these two measurements body mass index (BMI, kg/m^2^) was calculated.

### Biochemical analysis

A venous blood sample of 5 mL was obtained from all subjects after at least 12 hours of fasting. A part of the sample (2 mL) was collected into glass tubes and allowed to clot at room temperature, and used to determine serum lipid levels. Another part of the sample (3 mL) was transferred to tubes with anticoagulate solution (4.80 g/L citric acid, 14.70 g/L glucose, and 13.20 g/L tri-sodium citrate) and used to extract DNA. The levels of TC, TG, HDL-C, and LDL-C in samples were determined by enzymatic methods with commercially available kits, Tcho-1, TG-LH (RANDOX Laboratories Ltd., Ardmore, Diamond Road, Crumlin Co. Antrim, United Kingdom, BT29 4QY), Cholestest N HDL, and Cholestest LDL (Daiichi Pure Chemicals Co., Ltd., Tokyo, Japan); respectively. Serum ApoA1 and ApoB levels were detected by the immunoturbidimetric immunoassay using a commercial kit (RANDOX Laboratories Ltd.). All determinations were performed with an autoanalyzer (Type 7170A; Hitachi Ltd., Tokyo, Japan) in our Clinical Science Experiment Center [5,6].

### DNA amplification and genotyping

Genomic DNA was isolated from peripheral blood leukocytes using the phenol-chloroform method [7]. Genotyping of the *PPARD *+294T > C polymorphism was performed by polymerase chain reaction and restriction fragment length polymorphism (PCR-RFLP) [31]. PCR amplification was performed using 5'-CATGGTATAGCACTGCAGGAA-3' and 5'-CTTCCTCCTGTGGCTGCTC-3' (Sangon, Shanghai, People's Republic of China) as the forward and reverse primer pairs; respectively. Each amplification reaction was performed using 100 ng genomic DNA in 25 μL of reaction mixture consisting of 1.0 μL of each primer (10 μmol/L), 12.5 μL 2 × Taq PCR MasterMix (constituent: 0.1 U *Taq *polymerase/μL, 500 μM dNTP each and PCR buffer_)_. After initial denaturizing at 95°C for 5 min, the reaction mixture was subjected to 30 cycles of 45 s denaturation at 94°C, 45 s annealing at 62°C and extension 45 s at 72°C, followed by a final 8 min extension at 72°C. After electrophoresis on a 2.0% agarose gel with 0.5 μg/mL ethidium bromide, the amplification products were visualized under ultraviolet light. Then 5 U of *Bsl*I restriction enzyme was added directly to the PCR products (6 μL) and digested at 55°C overnight. After restriction enzyme digestion of the amplified DNA, the genotypes were identified by electrophoresis on 2.5% agarose gels and visualized with ethidium-bromide staining ultraviolet illumination. The genotypes were scored by an experienced reader blinded to epidemiological data and serum lipid levels.

### Diagnostic criteria

The normal values of serum TC, TG, HDL-C, LDL-C, ApoA1, ApoB levels, and the ratio of ApoA1 to ApoB in our Clinical Science Experiment Center were 3.10-5.17, 0.56-1.70, 0.91-1.81, 2.70-3.20 mmol/L, 1.00-1.78, 0.63-1.14 g/L, and 1.00-2.50; respectively. The individuals with TC > 5.17 mmol/L and/or TG > 1.70 mmol/L were defined as hyperlipidemic [5,6]. Hypertension was defined as an average systolic blood pressure of 140 mmHg or greater and/or an average diastolic blood pressure of 90 mmHg or greater, and/or self-reported pharmacological treatment for hypertension within the 2 weeks prior to the interview [38,39]. Normal weight, overweight and obesity were defined as a BMI < 24, 24-28, and > 28 kg/m^2^; respectively [40].

### Statistical analyses

Quantitative variables were expressed as mean ± standard deviation (serum TG levels were presented as medians and interquartile ranges). Qualitative variables were expressed as percentages. Allele frequency was determined via direct counting, and the standard goodness-of-fit test was used to test the Hardy-Weinberg equilibrium. Difference in genotype distribution between the groups was obtained using the chi-square test. The difference in general characteristics between nondrinkers and drinkers tested by the Student's unpaired *t*-test. The association of genotypes and serum lipid parameters was tested by analysis of covariance (ANCOVA). Sex, age, BMI, blood pressure, and cigarette smoking were adjusted for the statistical analysis. In order to evaluate the association of serum lipid parameters and genotypes (TT = 1, TC = 2, CC = 3), multiple linear regression analysis with stepwise modeling was also performed in the combined population of nondrinkers and drinkers, nondrinkers, and drinkers; respectively. All statistical analyses were done with the statistical software package SPSS 13.0 (SPSS Inc., Chicago, Illinois). A *P *value of less than 0.05 was considered statistically significant.

## Results

### General characteristics between nondrinkers and drinkers

Table 1 gives the general characteristics between nondrinkers and drinkers. The ratio of male to female, the levels of mean age, body height, weight, BMI, waist circumference, systolic blood pressure and diastolic blood pressure, and the percentages of subjects who smoked cigarettes were higher in drinkers than in nondrinkers (*P *< 0.001 for all). There was no significant difference in the levels of pulse pressure between the two groups (*P *> 0.05).

**Table 1:** The general characteristics and serum lipid levels in the nondrinkers and drinkers

Parameter	Nondrinker (n = 685)	Drinker (n = 497)	*t *(χ^2^)	*P*
Male/female	187/498	362/135	239.775	0.000
Age (years)	39.80 ± 16.37	44.93 ± 13.79	-5.691	0.000
Height (cm)	152.03 ± 7.75	155.34 ± 7.82	-7.181	0.000
Weight (kg)	51.05 ± 7.88	54.74 ± 8.21	-7.763	0.000
Body mass index (kg/m^2^)	22.04 ± 2.57	22.67 ± 2.91	-3.894	0.000
Waist circumference (cm)	69.80 ± 7.17	74.69 ± 7.09	-11.657	0.000
Systolic blood pressure (mmHg)	118.33 ± 16.94	122.79 ± 16.85	-4.475	0.000
Diastolic blood pressure (mmHg)	74.57 ± 9.82	77.70 ± 10.01	-5.357	0.000
Pulse pressure (mmHg)	43.76 ± 11.78	45.10 ± 12.74	-1.842	0.066
Cigarette smoking [n (%)]				
Nonsmoker	682 (89.2)	225 (45.3)		
< 20 cigarettes/day	34 (4.4)	120 (24.1)		
≥ 20 cigarettes/day	49 (6.4)	152 (30.6)	287.105	0.000
Alcohol consumption [n (%)]				
Nondrinker	685 (100.0)	-		
< 25 g/day	-	380 (76.6)		
≥ 25 g/day	-	116 (23.4)		
Total cholesterol (mmol/L)	4.53 ± 0.94	4.57 ± 1.07	-0.712	0.477
Triglyceride (mmol/L)	0.98 (0.59)	1.02 (0.77)	-2.923	0.003
HDL-C (mmol/L)	1.77 ± 0.47	1.83 ± 0.47	-2.102	0.036
LDL-C (mmol/L)	2.61 ± 0.72	2.56 ± 0.82	1.187	0.235
Apolipoprotein (Apo) A1 (g/L)	1.33 ± 0.27	1.41 ± 0.30	-4.928	0.000
ApoB (g/L)	0.87 ± 0.22	0.86 ± 0.23	0.806	0.420
ApoA1/ApoB	1.60 ± 0.49	1.77 ± 0.77	-4.600	0.000

HDL-C, high-density lipoprotein cholesterol; LDL-C, low-density lipoprotein cholesterol. The value of triglyceride was presented as median (interquartile range). The difference between the two groups was determined by the Wilcoxon-Mann-Whitney test.

### Serum lipid levels between nondrinkers and drinkers

The levels of serum lipid parameters between nondrinkers and drinkers are also shown in Table 1. The levels of TG, HDL-C, ApoA1, and the ratio of ApoA1 to ApoB were higher in drinkers than in nondrinkers (*P *< 0.05-0.001). There were no significant differences in the levels of TC, LDL-C and ApoB between the two groups (*P *> 0.05 for all).

### Results of electrophoresis and genotyping

After the genomic DNA of the samples was amplified by PCR and imaged by 2.0% agarose gel electrophoresis, the PCR products of 269 bp nucleotide sequences could be found in all samples. The genotypes identified were named according to the presence or absence of the enzyme restriction sites, when a T to C transversion at +294 locus of the *PPARD*. The presence of the cutting site indicates the C allele, whereas its absence indicates the T allele (cannot be cut). Thus, the TT genotype is homozygote for the absence of the site (band at 269 bp), TC genotype is heterozygote for the absence and presence of the site (bands at 269-, 167- and 102-bp), and CC genotype is homozygote for the presence of the site (bands at 167- and 102- bp). The genotype distribution was consistent with the Hardy-Weinberg equilibrium.

### Genotypic and allelic frequencies

The genotypic and allelic frequencies of *PPARD *+294T > C polymorphism in the nondrinkers and drinkers are shown in Table 2. The frequencies of TT, TC and CC genotypes were 56.0%, 36.4% and 7.6% in nondrinkers, and 57.2%, 38.0% and 4.8% in drinkers (*P *> 0.05); respectively. The frequencies of T and C alleles were 74.2% and 25.8% in nondrinkers, and 76.2% and 23.8% in drinkers (*P *> 0.05); respectively. There was also no significant difference in the genotypic and allelic frequencies between males and females in both groups (*P *> 0.05 for all).

**Table 2:** Genotypic and allelic frequencies of the PPARD +294T > C polymorphism in the nondrinkers and drinkers [n (%)]

Group	n	Genotype			Allele	
			
		TT	TC	CC	T	C
Nondrinker	685	384 (56.0)	249 (36.4)	52 (7.6)	1017 (74.2)	353 (25.8)
Drinker	497	284 (57.2)	189 (38.0)	24 (4.8)	757 (76.2)	237 (23.8)
χ^2^	-	3.697			1.138	
*P*	-	0.158			0.286	
Nondrinker						
Male	187	105 (56.2)	68 (36.4)	14 (7.4)	278 (74.3)	96 (25.7)
Female	498	279 (56.0)	181 (36.4)	38 (7.6)	739 (74.2)	257 (25.8)
χ^2^	-	0.004			0.003	
*P*	-	0.998			0.959	
Drinker						
Male	362	210 (58.0)	136 (37.6)	16 (4.4)	556 (76.8)	168 (23.2)
Female	135	74 (54.8)	53 (39.2)	8 (6.0)	201 (74.4)	69 (25.6)
χ^2^	-	0.712			0.599	
*P*	-	0.701			0.438	

### Genotypes and serum lipid levels

As shown in Table 3, the levels of TC in nondrinkers were different among the three genotypes (*P *= 0.01), the C allele carriers had higher serum TC levels than the C allele noncarriers. There was no significant difference in the remaining serum lipid parameters among the three genotypes in nondrinkers (*P *> 0.05 for all).

**Table 3:** Genotypes of the PPARD +294T > C polymorphism and serum lipid levels in the nondrinkers and drinkers

Group	Genotype	n	TC (mmol/L)	TG (mmol/L)	HDL-C (mmol/L)	LDL-C (mmol/L)	ApoA1 (g/L)	ApoB (g/L)	ApoA1/ ApoB
Nondrinker	TT	384	4.44 ± 0.91	0.98(0.57)	1.74 ± 0.44	2.57 ± 0.70	1.31 ± 0.26	0.86 ± 0.21	1.60 ± 0.43
	TC	249	4.62 ± 0.92	0.99(0.65)	1.80 ± 0.48	2.64 ± 0.71	1.35 ± 0.26	0.90 ± 0.24	1.59 ± 0.48
	CC	52	4.74 ± 1.12	0.89(0.57)	1.87 ± 0.54	2.77 ± 0.92	1.39 ± 0.30	0.91 ± 0.24	1.68 ± 0.88
*F*	-	-	4.629	0.217	2.667	2.301	2.545	2.810	0.734
*P*	-	-	0.010	0.897	0.070	0.101	0.079	0.061	0.480
Drinker	TT	284	4.57 ± 1.01	1.01(0.71)	1.83 ± 0.49	2.60 ± 0.82	1.41 ± 0.32	0.86 ± 0.23	1.77 ± 0.78
	TC	189	4.55 ± 1.16	1.02(0.89)	1.85 ± 0.46	2.47 ± 0.79	1.42 ± 0.28	0.86 ± 0.25	1.79 ± 0.69
	CC	24	4.74 ± 1.08	1.26(0.93)	1.68 ± 0.43	2.73 ± 0.97	1.38 ± 0.31	0.93 ± 0.28	1.67 ± 0.94
*F*	-	-	0.355	3.836	1.365	2.189	0.184	1.205	0.300
*P*	-	-	0.702	0.147	0.256	0.113	0.832	0.301	0.741

TC, total cholesterol; TG, triglyceride; HDL-C, high-density lipoprotein cholesterol; LDL-C, low-density lipoprotein cholesterol; ApoA1, apolipoprotein A1; ApoB, apolipoprotein B; ApoA1/ApoB, the ratio of apolipoprotein A1 to apolipoprotein B. The value of TG was presented as median (interquartile range). The difference among the genotypes was determined by the Kruskal-Wallis test.

The levels of all seven lipid traits in drinkers were not different among the three genotypes (P > 0.05 for all).

### Interactions between genotypes and alcohol on serum lipid parameters

The interaction between *PPARD *+294T > C genotypes and alcohol consumption on serum TC levels (*F *= 0.706, *P = *0.494) and the remaining serum lipid parameters was not detected by using a factorial regression analysis after controlling for potential confounders.

### Correlation between serum lipid parameters and genotypes

Multiple linear regression analysis showed that serum TC, HDL-C, LDL-C, ApoA1, and ApoB levels were correlated with genotypes in drinkers but not in nondrinkers (*P *< 0.05-0.01; Table 4).

**Table 4:** Correlation between serum lipid parameters and the PPARD +294T > C genotypes in the nondrinkers and drinkers

Lipid	Relative factor	Unstandardized coefficient	Standard error	Standardized coefficient	*t*	*P*
Nondrinker plus drinker						
TC	Genotype	0.123	0.046	0.080	2.833	0.005
ApoB	Genotype	0.025	0.010	0.071	2.527	0.012
Drinker						
TC	Genotype	0.167	0.055	0.113	3.058	0.002
HDL-C	Genotype	0.068	0.028	0.092	2.440	0.015
LDL-C	Genotype	0.089	0.042	0.078	2.110	0.035
ApoA1	Genotype	0.036	0.016	0.086	2.279	0.023
ApoB	Genotype	0.030	0.013	0.086	2.336	0.020

## Discussion

The present study showed that the levels of serum TG, HDL-C, ApoA1, and the ratio of ApoA1 to ApoB were higher in drinkers than in nondrinkers. There were no significant differences in the levels of TC, LDL-C and ApoB between the two groups. These results suggest that the great majority of our study populations are beneficial from alcohol consumption. Low to middle amounts of alcohol when taken on a regular basis have been shown to protect against CAD and death [12,13]. A moderate intake of alcohol is associated with protection against CAD, probably due in part to a dose-dependent increase in HDL-C and ApoA1 levels [14-17]. According to a previous meta-analysis, a daily dose of 30 g alcohol results in an average HDL-C level rise of 3.99 mg/dl, and an ApoA1 level rise of 8.82 mg/dl [14]. The harmful effects of heavy alcohol consumption on serum lipid profiles may be due to an increase in plasma TG levels [14,18]. In the previous meta-analysis, 30 g of alcohol daily was associated with a plasma TG increase of 5.69 mg/dl [14]. The alcohol intake of 60 g/day increases the TG levels by about 0.19 mg/dl per 1 gram of alcohol consumed [18]. The effects of alcohol consumption on LDL-C are inconsistent. A recent study in older Italian subjects (65-84 years old) has found that alcohol intake increases serum LDL-C levels [41]. Another recent study of Turks also found increases in LDL-C, as well as in ApoB and TG, with alcohol in men, while women had decreased TG and no change in LDL-C or ApoB with alcohol [42]. A decrease in LDL-C with increased alcohol intake has also been reported in some studies, but this effect is less consistent and probably depends on the combination of one or more unmeasured factors [17].

The genotypic and allelic frequencies of *PPARD *+294T > C polymorphism were different in diverse populations. Several previous studies have showed that the frequency of the rare allele (+294C) was 18.3% in Russian endurance-oriented athletes and 12.1% in controls (*P *< 0.0001) [43], 32.0% in Tunisian CAD patients and 18.9% in healthy volunteers (*P *= 0.001) [32], and 30.8% in Chinese CAD patients and 19.5% in normal controls (*P *< 0.05) [33]. Other studies, however, showed that there was no difference in its frequency between the patients with type 2 diabetes and the non-diabetic controls (18.7% vs 19.2%) [35], or among the patients with metabolic syndrome, essential hypertension and type 2 diabetes [26]. In the present study, we showed that the frequency of +294C alleles was 25.8% in nondrinkers, and 23.8% in drinkers (*P *> 0.05). There was no significant difference in the genotypic and allelic frequencies between males and females in both groups. However, the frequency of *PPARD *+294C allele was higher in our study population than in 543 healthy 50-year-old-men (15.6%) from the northern part of the greater Stockholm area [24], in normal controls (19.5%) from Chinese Anhui Province [33], in healthy Tunisian population (18.9%) [32], and in non-diabetic Germany controls (19.2%) [35]; but it was lower than in Tunisian CAD patients (32.0%) [32] and Chinese CAD patients (30.8%) [33]. These results indicate that the prevalence of the C allele variants of *PPARD *+294T > C polymorphism may have an ethnic or disease specificity.

The association of *PPARD *+294T > C polymorphism and serum lipid levels is inconsistent. Skogsberg et al. [24] demonstrated that homozygotes for the rare C allele had a higher LDL-C concentration than homozygotes for the common T allele. There were no associations with the HDL-C levels. In another study in Scottish men, they found that the +294C allele did not influence LDL-C concentrations but was associated with lower HDL-C levels [25]. Aberle et al. [27] also showed a highly significant association between the rare C allele and lower HDL-C levels in dyslipidemic female subjects. In addition, metabolic syndrome patients with CC genotype had significantly higher TC and LDL-C levels than those with TT and TC genotypes [26]. The *PPARD *+294T > C polymorphism was associated with HDL-C and was dependent on sex among subjects with and without type 2 diabetes [29]. The risk variant of *PPARD *+294T > C marker was associated with higher LDL-C and increased serum TC [31]. However, Gouni-Berthold *et al*. [35] found that the presence of the C allele had no effect on TG, HDL-C, and LDL-C levels, both in diabetic and non-diabetic German controls, or both in men and in women. The same result was found by Jguirim-Souissi et al. [32] both in CAD patients and healthy controls. In the present study, we showed that the levels of TC in nondrinkers were different among the three genotypes, the C allele carriers had higher serum TC levels than the C allele noncarriers. But the levels of all seven lipid traits in drinkers were not different among the three genotypes. Serum TC, HDL-C, LDL-C, ApoA1, and ApoB levels were correlated with genotypes in drinkers but not in nondrinkers. These results suggest that the effects of *PPARD *+294T > C polymorphism on serum lipid levels are different between nondrinkers and drinkers.

The interactions between *PPARD *+294T > C polymorphism and alcohol consumption on serum lipid levels have not been previously explored. In a previous study, Brand-Herrmann *et al*. [9] showed that alcohol consumption modulates the relation between the PPAR-gamma 2 (PPARG) Pro12Ala and HDL-C. They randomly recruited 251 nuclear families (433 parents and 493 offspring) in the framework of the European Project on Genes in Hypertension study and genotyped 926 participants in whom all serum lipid variables and information on alcohol consumption were available for PPAR-gamma 2 Pro12Ala. The results showed that the Ala12 allele was more frequent in Novosibirsk (17%) than in Cracow (12%) and Mirano (11%, *P *< 0.01). Italian offspring carrying the Ala12 allele had higher serum HDL-C than noncarriers (*P *< 0.05). HDL-C levels were on average 0.086 mmol/L (*P *= 0.001) higher in drinkers than in nondrinkers. As compared with Pro12 homozygotes, Ala12 allele carriers consuming alcohol had higher serum total and HDL-C, with the opposite trend occurring in nondrinkers. This genotype-alcohol interaction was independent of the type of alcoholic beverage and more pronounced in moderate than in heavy drinkers. In the present study, however, we found no interaction between the *PPARD *+294T > C genotypes and alcohol consumption on serum lipid levels in the drinkers. These findings suggest that increased levels of TG, HDL-C, ApoA1, and the ratio of ApoA1 to ApoB in drinkers were not influenced by the interactions of *PPARD *+294T > C polymorphism and alcohol consumption. The effect of different kinds of wine on serum lipid profiles is not well known. In the present study, 90% of the wine drunk by the subjects was corn wine, rice wine or rum, in which the alcohol content is low. Thus, the effects of different kinds of alcohol consumption on serum lipid levels still need to be determined [44,45].

## Conclusion

The present study shows that the levels of TG, HDL-C, ApoA1, and the ratio of ApoA1 to ApoB were higher in drinkers than in nondrinkers. There were no significant differences in the levels of TC, LDL-C and ApoB, and the genotypic and allelic frequencies of *PPARD *+294T > C between nondrinkers and drinkers. The levels of TC in nondrinkers were different among the three genotypes, the C allele carriers had higher serum TC levels than the C allele noncarriers, whereas the levels of all seven lipid traits in drinkers were not different among the three genotypes. The interaction of *PPARD *+294T > C genotypes and alcohol consumption on serum TC levels and the remaining serum lipid parameters was not detected in the drinkers. Multiple linear regression analysis showed that serum TC, HDL-C, LDL-C, ApoA1, and ApoB levels were correlated with genotypes in drinkers but not in nondrinkers. These results suggest that the great majority of our study populations are beneficial from alcohol consumption. But there is no interaction between the *PPARD *+294T > C genotypes and alcohol consumption on serum lipid levels in the drinkers.

## Competing interests

The authors declare that they have no competing interests.

## Authors' contributions

XLW participated in the design, helped to carry out the genotyping, and drafted the manuscript. LM undertook genotyping. DFW collaborated to the genotyping and performed the statistical analyses. RXY conceived the study, participated in the design, carried out the epidemiological survey, collected the samples, and helped to draft the manuscript. All authors read and approved the final manuscript.
